# Human procurement of meat from lion *(Panthera leo)* kills: Costs of disturbance and implications for carnivore conservation

**DOI:** 10.1371/journal.pone.0308068

**Published:** 2024-08-14

**Authors:** Paula A. White, Laura D. Bertola, Kennedy Kariuki, Hans H. de Iongh

**Affiliations:** 1 Center for Tropical Research, Institute of the Environment and Sustainability, University of California Los Angeles, Los Angeles, California, United States of America; 2 Department of Biology, University of Copenhagen, Copenhagen, Denmark; 3 Leo Foundation, Wageningen, The Netherlands; 4 Institute of Environmental Sciences, Leiden University, RA Leiden, The Netherlands; Cheetah Conservation Fund, Namibia University of Science and Technology, NAMIBIA

## Abstract

In Africa, humans and large carnivores compete over access to resources, including prey. Disturbance by humans to kills made by carnivores, often for purposes of obtaining all or portions of the carcass, constitutes a form of human-wildlife conflict. However the occurrence of this practice, known as human kleptoparasitism, and its impact on carnivores has received little scientific attention. We obtained expert opinions from African lion researchers and stakeholders via a standardized questionnaire to characterize the geographic extent and frequency of human kleptoparasitism as it occurs in modern times. Our survey found modern human kleptoparasitism on kills made by lions, and possibly other large carnivores in Africa, to be geographically more widespread than previously reported. Meat lost to humans requires carnivores to hunt and kill additional prey thereby causing stress, increasing their energetic costs and risks of natural injury, and exposing them to risk of direct injury or death from human usurpers. Because of their conspicuous behaviors and tendency towards killing large-bodied prey, lions are particularly susceptible to humans detecting their kills. While human kleptoparasitism was geographically widespread, socio-economic factors influenced the frequency of occurrence. Prey type (wild game or domestic livestock) influenced human attitudes towards meat theft; ownership allows for legal recovery of livestock carcasses, while possessing wild game meat is mostly illegal and may incur penalties. Meat theft was associated with other illegal activities (i.e., illegal mining) and most prevalent among people of low income, including underpaid game scouts. Despite quantifiable costs to carnivores of human disturbance to their kills, the majority of experts surveyed reported a lack of knowledge on this practice. We propose that human disturbance at kills, especially loss of prey through human kleptoparasitism, constitutes an important anthropogenic threat that may seriously impact energy budgets of individual lions and other scavengers when meat and carcasses are removed from the ecosystem, and that the costs incurred by carnivores warrants further investigation.

## Introduction

Carnivores incur energetic costs and risk injury when hunting and killing their prey [1]. Each stage of a hunt e.g., locating, stalking or chasing, and killing prey requires considerable energy expenditure [2–8]. Prey pursuit and capture entails physical risks to predators [9], including the possibility of being injured [10] or killed [11, 12]. Following a successful hunt, a carnivore may face the added cost and risk of defending its prey from being stolen (kleptoparasitized [13]) by conspecifics, other carnivore species [14–18], or by humans [19, 20]. While considerable attention has been paid to humans deliberately killing carnivores (e.g., trophy hunting [21–23], and lethal removal as a form of management [24]), non-lethal impacts have received far less consideration [25–28].

Human activities such as ecotourism viewing can disturb and stress carnivores [25, 29, 30], including while the animals are hunting [31–32]. Humans also target carnivore kills to obtain meat for personal consumption and to sell or trade [19, 20, 33–36].

Although early hominids are believed to have, at times, hunted game [37], the practice of early hominids obtaining meat from carnivore kills, referred to as human kleptoparasitism, has been well-documented [38–41]. Human kleptoparasitism is known to still occur in modern times (i.e., during the past 60 years) [19, 20, 33, 34, 36]. In their examination of data from human-carnivore conflicts in Uganda between the years 1923–1994, including human kleptoparasitism, Treves and Naughton-Treves (1999) highlighted the interlinked evolutionary histories of humans and early hominids that shared their habitats with large carnivores. However, to date, the geographic scale, frequency, and the potential impact on carnivores of the modern practice of human kleptoparasitism have not been adequately investigated [20]. A paucity of reports 35 years ago led Sunquist and Sunquist (1989) to conclude that while human kleptoparasitism appeared to pose no significant threat to individual carnivores in the short-term, repeated losses could negatively impact carnivore populations in the long-term. Lacking an in-depth exploration of the modern practice, it is likely that both the costs to individual carnivores, and the population-wide effects, have been underestimated.

Humans are now considered a “super-predator” [42], and human disturbance in the proximity of kills has been shown to induce fear in large carnivores [43–45], likely resulting in stress, reducing the overall time spent at the carcass [44], and reducing the total amount of meat consumed [36, 46, 47]. Even a perceived risk of encountering humans may cause carnivores to alter their behaviors and movements [27, 44, 45, 48], including abandoning their kills [49], or shifting to increased nocturnality [50].

Loss of kills to humans increases the frequency at which a carnivore must hunt [36, 43, 47], thereby increasing the costs and risks for an animal to obtain the equivalent amount of energy i.e., meat [44]. For carnivores that return to their kill following human disturbance, loss of a partial or complete carcass decreases or eliminates the amount of meat remaining for subsequent feeding bouts [33], reducing the energy gained per kill [44]. Removal of carrion also reduces or eliminates food resources for scavengers [36] potentially disrupting scavenger communities and associated ecosystem services e.g., nutrient cycling [51–54].

Humans that actively chase carnivores from their kills to obtain meat (confrontational scavenging) may injure carnivores, or may themselves be injured or killed in the process [19, 55]. Carnivores that injure humans in defense of their kills may subsequently be destroyed as problem animals [19, 55]. Additionally, once kills have been located, humans may poison carcasses [56–58] to kill carnivores and scavengers under the auspices of animal control, in retaliation for livestock depredation [59, 60], or to obtain body parts for illegal trade [61, 62].

Despite this broad range of known impacts, our understanding of the extent and frequency of modern human kleptoparasitism is lacking. The few previous reports available have focused primarily on anecdotal or regional accounts [19, 20, 33, 34]. However, other factors e.g., geographic location of the kill within or outside of protected area boundaries, legal ramifications of possessing game meat, and cultural or religious taboos, may influence the occurrence of human kleptoparasitism.

Given that the majority of modern threats to large carnivores are anthropogenic [63–65], and that anthropogenic threats are likely to be cumulative, further investigation into the potential impacts of modern human kleptoparasitism on carnivores is warranted.

To address this data gap, we conducted a questionnaire survey among lion experts and stakeholders with knowledge of the African lion, *Panthera leo*, in the current African range states to elucidate the geographic extent, frequency, and socio-economic drivers of modern human kleptoparasitism. Due to their conspicuous social behaviors and reliance on large-bodied prey [66], lions may be particularly vulnerable to being detected on their kills [67]. While our survey focused on the kills made by lions, respondents were asked about the occurrence of human kleptoparasitism on other large carnivores in their areas i.e., leopard, *Panthera pardus*, spotted hyena, *Crocuta crocuta*, wild dog, *Lycaon pictus*, cheetah, *Acinonyx jubatus*.

This study represents the first effort to systematically characterize and assess modern human kleptoparasitism across the range of the African lion, and to illustrate the suite of potential costs faced by carnivores exposed to this practice.

## Methods

We developed a questionnaire survey with a goal of quantifying the geographic extent, rate of occurrence, trends, and other associated factors e.g., legality, cultural or religious taboos, of humans taking meat from the kills of large carnivores in sub-Saharan Africa. To prospectively recruit participants, we distributed a pre-structured questionnaire (S1 Appendix) among lion experts in the African Lion Working Group (ALWG), a working group of approximately 90 members under auspices of the IUCN Cat Specialist Group. The ALWG represents active lion researchers, and other stakeholders, whose work may not involve field studies. Thus, we did not anticipate receiving responses from every ALWG member. Additionally, we circulated the survey among approximately 35 other stakeholders with knowledge of wild carnivores (with a focus on *P*. *leo*) i.e., conservation organizations, lodge owners, and professional hunters in lion range states. The goal was to query lion experts’ and stakeholders’ existing knowledge i.e., direct observations, received reports, and opinions of the practice within their geographic area(s). Although some survey recipients did not conduct scientific studies per se, the geographic area described in a respondents’ survey is hereafter referred to as their study area.

On 31 May 2020, we circulated the questionnaire in the text of an e-mail and as an attached document with a request for respondents to reply through whatever means was most convenient. We sent a follow-up reminder on 15 June 2020, and set a cut-off date of 2 July 2020 to receive responses. If respondents had knowledge about more than one study area, we requested that they complete one questionnaire for each study area. In addition to completed questionnaires, some respondents also provided comments via e-mail; others provided only e-mail comments but did not complete the questionnaire. Some respondents who completed the survey did not answer every question, thus reported sample sizes vary. Additionally, some research teams designated one person to reply on behalf of the team with information from their study area; such replies were tallied as one respondent. All answers, whether obtained from questionnaires or in e-mail format, were incorporated into the results to the extent possible. Apart from maintaining the geographic context (country) of the data received, responses were combined and the specific locations of the study areas, as well as the identities of the responding individuals, were kept confidential.

The pre-structured survey form contained twelve questions focused on whether kleptoparasitism by humans had been observed or reported to occur in the respondent’s study area, how information was obtained, and details of the practice e.g., how often and by whom (S1 Appendix). Further, we examined whether the kleptoparasitism events involved wild prey or livestock, and how prey type influenced human attitudes and responses. Respondents were asked how long they had worked in the study area (tenure), and, whether in their expert opinion, kleptoparasitism represented a potential threat to lions in their study area (Question #8) or across the species’ range (Question #9). For respondents who completed surveys for more than one study area, we counted their answer to Question #9 only once regardless of how many surveys they completed. All other answers were tallied as representing individual responses. Because the answer options to the frequency of occurrence were presented as a range of values e.g., 1–5, 6–10, etc., (S1 Appendix), we used the average value within each option when considering frequency of occurrence on respondents’ tenure. For example, for option a) 1–5 times/year, we used a value of 2.5 times/year when examining frequency of occurrence on tenure.

We performed descriptive statistics on the different responses to each question, and performed a Welch two-sided t-test to examine whether knowledge of the presence of human kleptoparasitism in a study area was influenced by the respondents’ tenure in that area. We also performed chi-square tests of independence to examine whether a respondent’s knowledge of kleptoparasitism in their study area influenced their opinion regarding whether or not kleptoparasitism represented a potential conservation threat to lions across the species’ range. We considered a result statistically significant at p < 0.05.

### Ethics statement

We followed the checklist of the Science Faculty of Leiden University and concluded that application with the Ethics Committee did not apply for our survey. All respondents completed the survey voluntarily and provided written informed consent via survey(s) or e-mail for the data to be used for this purpose. All responses were treated as confidential. The completed PLOS ONE Human Subjects Research Checklist was included as part of this submission.

### Inclusivity in global research

Additional information regarding the ethical, cultural, and scientific considerations specific to inclusivity in global research is included in the Supporting Information (S1 Checklist).

## Results

**Fig 1:**
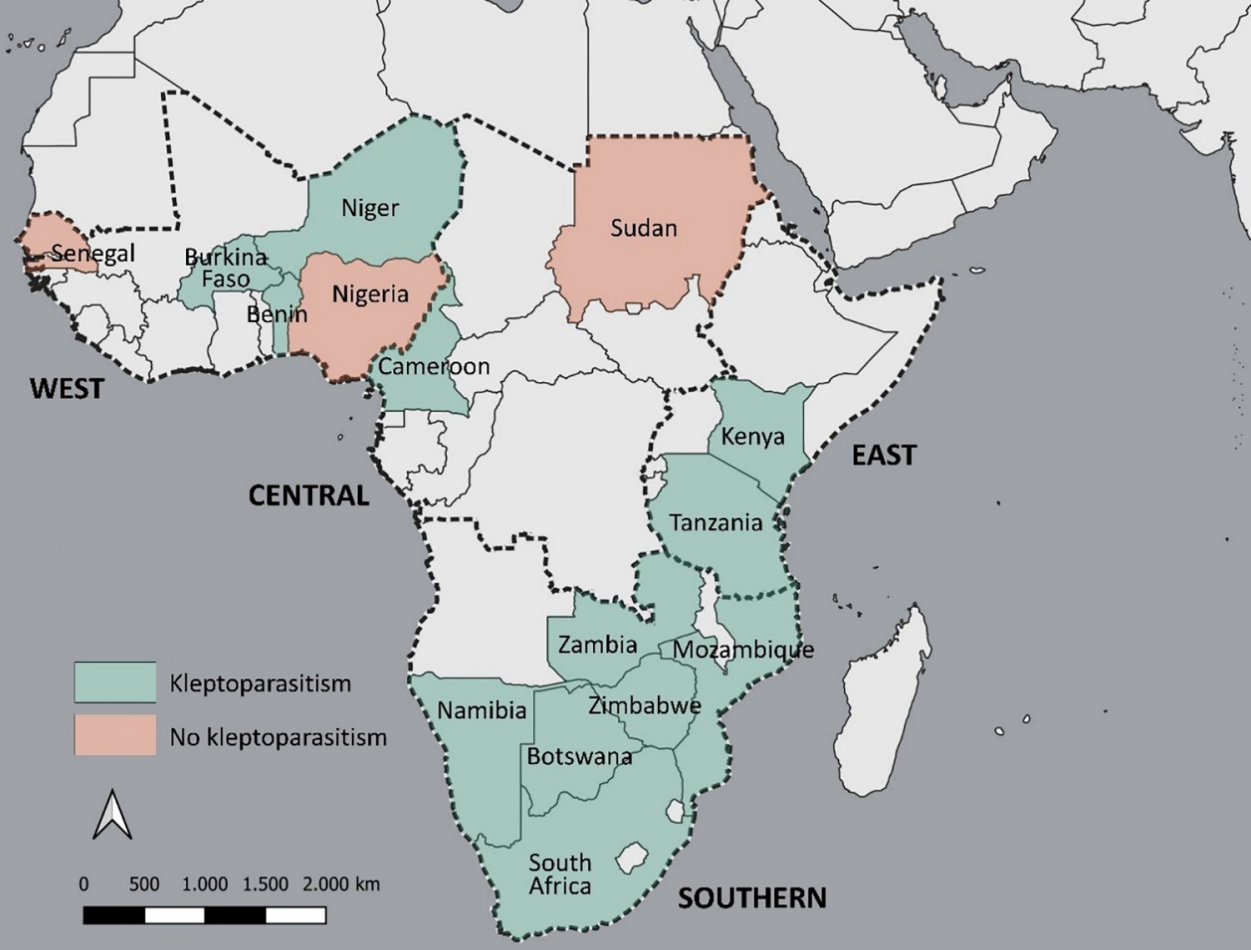
Map of the African lion range states surveyed for modern human kleptoparasitism. Highlighting indicates countries in which occurrence (green) or no occurrence (brown) was reported. The geographic regions of West, Central, East, and Southern Africa are demarked by dotted black lines. Figure generated with QGIS software by author Laura Bertola using layers (e.g., country borders) which are copyright-free to re-purpose for non-commercial use.

We received 56 completed questionnaires from 33 individual respondents (14 respondents completed questionnaires for more than one study area). An additional 5 respondents provided e-mailed comments but did not complete a survey. The 38 total respondents were comprised of 31 ALWG members and 7 stakeholders (response rate 34% and 20%, respectively). Thus, our results represent experts’ knowledge of extant lion populations from 56 studies located in 15 of 22 African lion range states (Fig 1). The 15 surveyed range states include all resident lion populations in West Africa and nearly all East and Southern African range states that, combined, account for > 95% of Africa’s lions [68] (S1 Table). The occurrence of human kleptoparasitism on the kills of wild carnivores was reported in 37 of the 56 questionnaires (66%) received, confirming modern occurrence of the practice in 12 of the 15 range states surveyed (Fig 1; S1 Table).

The number of years (tenure) spent by individual respondents in their respective study areas ranged from 1 to 30 years (*X* = 11.9 years). There was no significant difference in tenure between respondents who reported occurrence of kleptoparasitism in their area (*X* = 10.8 years) and those who reported no occurrence (*X* = 11.6 years, Welch two-sample t-test (26.9) = 0.3, p = .76). There was no significant association between a respondent having reported human kleptoparasitism within a study area and whether, in their expert opinion, this practice represented a threat to lions across the species’ range (Pearson’s chi-square test χ2 (1, N = 46) = 0.004, p = .94).

A respondent’s knowledge of whether kleptoparasitism occurred in their study area was most often gained through reliable reports (40%) and direct observation (35%), followed by local gossip (25%) (Table 1). A reliable report was subjectively defined as a field assistant, wildlife officer, professional hunter, or conservation organization who themselves had directly observed the practice and subsequently relayed that information to the survey respondent.

**Table 1:** Respondents’ source for knowledge on the occurrence of kleptoparasitism, strategy for obtaining meat from kills, who procured meat, and nature of activity.

*Survey Question*			
*(number of responses/total sample size; %)*			
Source of knowledge of kleptoparasitism	Strategy for obtaining meat from kills	Who procured meat	Nature of activity
Reliable reports (22/55; 0.40)	Actively chase (26/52; 0.50)	Local residents (33/56; 0.59)	Opportunistic (35/40; 0.88)
Direct observation (19/55; 0.35)	Scavenge (19/52; 0.36)	Game scouts (16/56; 0.29)	Deliberate targeting (4/40; 0.10)
Local gossip (14/55; 0.25)	Other e.g., playbacks (7/52; 0.13)	Non-residents (7/56; 0.12)	Unknown (1/40; 0.3)

Respondents indicated that in half (50%) of all reported occurrences, humans actively chased lions from kills; 36% of the time humans scavenged meat from kills when lions were not present. An additional 13% of cases involved other strategies of humans obtaining meat, including responding to playbacks broadcast by researchers to attract carnivores (Table 1). In the latter case, because the playback imitated the sounds of a dying prey animal, it could be assumed that the intention of the approaching humans was to obtain meat by actively chasing the lions off of a fresh kill. However, because this was an assumption, these cases were grouped under “Other”.

Meat procurement was reported to be undertaken mostly by local residents (59%), or game scouts (29%). Game scouts are here defined as employees working for a wildlife authority or private organization who are trained to combat wildlife crimes and whose duties include apprehending poachers, confiscating wire snares and firearms, etc. A smaller percentage (12%) of cases involved non-residents (people who did not reside full-time in the community) i.e., transient or nomadic people, refugees, or seasonal workers (Table 1). The activity was reported as being largely opportunistic (88%), although a few respondents (10%) reported that the act involved deliberate targeting and following lions that were engaged in hunting (Table 1).

**Fig 2:**
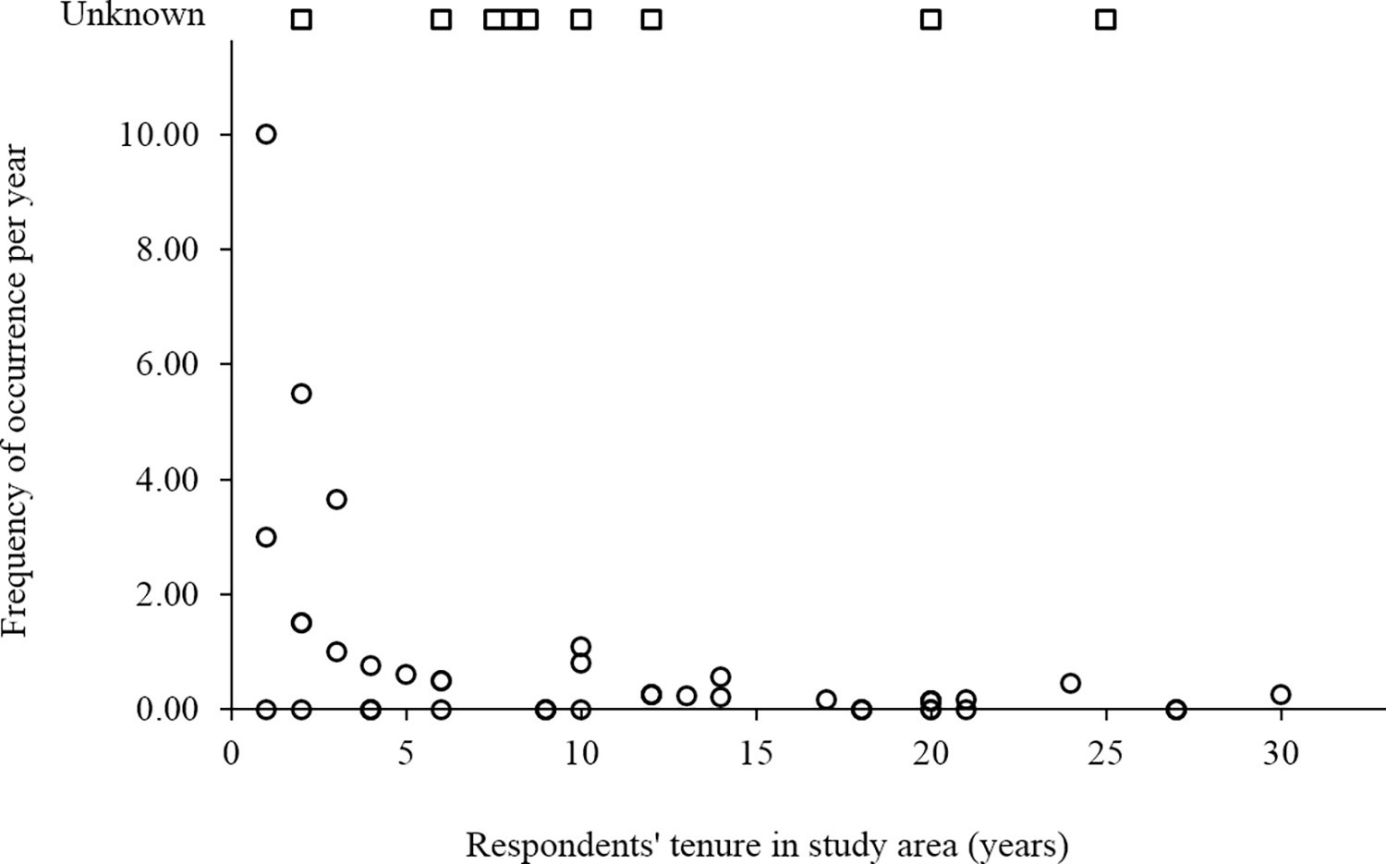
Frequency of occurrence of kleptoparasitism in relation to respondents’ tenure. Square points show the tenure of nine respondents who reported kleptoparasitism occurring in their study area at unknown frequency.

Of those respondents reporting kleptoparasitism, most (53%) had encountered human kleptoparasitism in their study area 1–5 times; others reported 5–10 occurrences (8%), or more than 10+ occurrences (14%). An additional 25% of respondents were aware of kleptoparasitism occurring in their study area, but did not know how often it occurred. Because length of tenure varied by individual respondent, we calculated frequency of occurrence per year of respondents’ tenure so that rates were comparable across study areas and time periods (Fig 2).

Most respondents (49%) did not know whether the activity was increasing, stable, or decreasing; 30% reported steady occurrence of the practice, with smaller numbers reporting a decrease (14%) or increase (8%) during their tenure (Table 2).

**Table 2:** Trend of occurrence, level of perceived threat within a study area and across the species’ range, cultural/religious significance, and prey type involved with human kleptoparasitism on lion kills.

*Survey Question*				
*(number of responses/total sample size; %)*				
Trend of occurrence	Level of perceived threat (within study area)	Cultural/Religious significance	Prey type	Perceived as threat across species’ range
*Unknown (18/37; 0*.*49)*	No serious threat (23/37; 0.62)	Neutral (15/37; 0.41)	Wild game	No
			(20/37; 0.54)	(23/32; 0.72)
Steady occurrence (11/37; 0.30)	*Insufficient knowledge (8/37; 0*.*22)*	Acceptable (13/37; 0.35)	Livestock	Yes
			(17/37; 0.46)	(9/32; 0.28)
Decreasing occurrence	Possible threat/Potential negative impact	*Insufficient knowledge (7/37; 0*.*19)*		
(5/37; 0.14)				
	(6/37; 0.16)			
Increasing occurrence		Religious taboo		
		(1/37; 0.03);		
(3/37; 0.08)		Occurs despite taboo		
		(1/37; 0.03)		

Results shown in italics indicate proportion of respondents stating insufficient knowledge on the topic.

Most (62%) respondents perceived human kleptoparasitism as representing no serious threat i.e., having no negative impact to lions within their study area. However, 16% of respondents perceived human kleptoparasitism as a possible threat with a potential to negatively impact lions within their study area (Table 2); 22% reported insufficient knowledge on the topic.

From a cultural standpoint, the majority reported the practice of obtaining meat from carnivore kills as being neutral (41%), or socially acceptable (35%). Two respondents reported that kleptoparasitism went against local religious beliefs, although one indicated that the practice occurred despite the taboo. The remaining respondents (19%) reported insufficient knowledge on the topic (Table 2).

Results showed that human kleptoparasitism was geographically widespread (Table 3). Prey type (wild game vs domestic livestock) was an important component in the likelihood of humans taking kills. Of the 37 respondents, slightly more than half (54%) reported carnivores losing wild game to humans, while the remaining 46% reported that humans only took the meat if carnivores had killed livestock.

**Table 3:** Trend, perceived threat of kleptoparasitism within study areas, and prey type by region in surveyed range states where human kleptoparasitism was reported.

	*Survey Question*		
	Trend	Perceived threat in study area Possible/Unlikely/Unk (n)	Prey type
	Increase/Stable/Decrease/Unk (n)		Wild game/Livestock (n)
*Region*			
**West & Central Africa**	0 / 0.17 / 0 / *0*.*83* (6)	0.17 / 0.67 / *0*.*17* (6)	0.83 / 0.17 (6)
**East Africa**	0.07 / 0.33 / 0.07 / *0*.*47* (15)	0.07 / 0.60 / *0*.*33* (15)	0.43 / 0.57 (14)
**Southern Africa**	0.12 / 0.25 / 0.19 / *0*.*44* (16)	0.25 / 0.62 / *0*.*12* (16)	0.53 / 0.47 (17)

Results shown in italics indicate proportion of respondents stating insufficient knowledge on the topic.

Because livestock (numbers, species) and culture/religious practices vary regionally, we also considered trend, perceived threat within a study area, and prey type by consolidating responses into one of three regions: West and Central Africa (regions combined due to small sample sizes), East Africa, and Southern Africa. In West and Central Africa, livestock was the predominant prey type involved in human kleptoparasitism, whereas in both East and Southern Africa, humans took wild game and livestock prey in close to equal measures (Table 3).

In all regions, a high percentage of respondents reported insufficient knowledge regarding trends of occurrence and whether, in their expert opinion, the practice represented a threat to carnivores (Tables 2 and 3). Regionally, a greater percentage of respondents from Southern Africa (25%) perceived the practice as possibly representing a threat to lions within their study areas, compared to respondents from West and Central (17%) or East (7%) Africa (Table 3). More than one-quarter (28%) of those responding perceived human kleptoparasitism on lion kills as representing a threat across the species’ range (Table 2).

Human kleptoparasitism on kills made by other large carnivore species also was investigated. More than half (20/37, 54%) of respondents reported insufficient knowledge on species other than lion. However, because the survey targeted experts and stakeholders with knowledge specifically on lions, and because not all other species of large carnivores occur throughout the entire lion range, the number of reports of meat taken from other species are not directly comparable. In summary, twelve respondents had knowledge of leopard losing kills to humans, seven reported occurrence of meat theft from spotted hyena, while fewer (4) and (3) respondents reported meat theft from cheetah and wild dog, respectively.

## Discussion

For millions of years, large carnivores have competed over access to carcasses, adapting their behaviors and social systems to maximize benefits while reducing costs of intra and interspecific kleptoparasitism [15, 66, 69–72]. Concurrently, early hominids are believed to have included meat in their diet both from scavenging and actively chasing carnivores off kills [41, 73–76]. This practice, which may have represented a survival strategy for ancient humans, still exists in present-day Africa as a means for people to obtain animal protein. Traditional hunters, such as the Hazda, still obtain meat by scavenging from lions [73, 77, 78]. Schaller (1972) [79] demonstrated how readily humans can displace lions to scavenge their kills [80]; other cases of modern human kleptoparasitism have been documented [19, 20].

If large carnivores have evolved to fight for possession of their kills, sometimes losing that battle to competitors, why should modern human kleptoparasitism be a cause for concern?

The dynamics between modern humans and large carnivores have changed. The majority of modern threats to large carnivores are anthropogenic e.g., habitat loss and alteration [64], reduction of prey populations [63], and retaliatory killing [65]. Growing human populations and associated development adjacent to shrinking protected areas is increasing human-wildlife conflicts [81, 82] including direct competition for wild prey [83, 84], although not all studies agree that human-carnivore conflict is increasing [85] (but see [86]). As “super-predators” [42], humans now increasingly impact large carnivores inducing fear [43, 44], causing stress [25, 29], and altering carnivore behavior, activity patterns, and movements [27, 43, 44, 45, 48, 50, 87, 88].

Consequences of human disturbance may be most severe when carnivores are feeding [26, 32], resulting in a reduction of the total amount of meat consumed [36, 46, 47]. The mere perception of human threat i.e., the sound of humans conversing, caused pumas, *Puma concolor*, to shorten their feeding periods [44]. Carnivores that lose substantial portions of their kills must increase their hunt frequency and kill additional prey to replace the resources lost [36, 43, 44, 47]. Stress, energy expenditure, and risk of physical injury associated with replacing lost resources may especially impact vulnerable individuals e.g., pregnant or nursing females [89], young cubs, and animals in poor condition, as well as the most vulnerable populations e.g., lions in West and Central Africa where prey densities are very low and, thus, the energetic costs of replacing lost prey are especially high [90, 91]. Moreover, the resultant increase in predation can potentially exacerbate human-wildlife conflict if carnivores target livestock to replace their lost kills. Ultimately, sub-lethal physiological effects of human disturbance may reduce individual fitness or survival [92, 93], and human-induced behavioral changes may alter the functional role of large carnivores as apex predators [44, 94].

Our study found modern human kleptoparasitism to be geographically widespread in sub-Saharan Africa. Surveyed experts confirmed the modern practice in 12 countries in Africa that constitute the majority of extant lion populations [68]. This represents the minimum geographic extent; kleptoparasitism may occur in lion range states not surveyed in our study. Our results may also contain reporting bias if those aware of the practice were more likely to report it; this applies both to survey recipients and to local people. Further, the likelihood of local people detecting and reporting kleptoparasitism to our experts may have been greater in areas of higher human density. Conversely, reluctance of local people to disclose the activity may have resulted in underestimates of occurrence. Thus, it is unlikely that non-response at either the expert or local level directionally biased our results. Nearly one-half of the survey respondents cited insufficient knowledge as to whether human kleptoparasitism was increasing, stable, or decreasing, underscoring a dearth of information on the modern practice.

Human detection of kills was largely opportunistic. However, once a kill was found, humans actively chased carnivores off of the carcass 50% of the time. More than half of all reported cases involved humans taking kills of wild game. The taking or possession of game meat is a largely illegal activity in most African countries [95], and the risk of disciplinary action may prompt discretion among those involved. This includes enforcement personnel; in about one quarter of the cases, anti-poaching game scouts were reported to be secondary participants, often keeping confiscated meat for themselves. Similarly, Treves and Naughton-Treves (1999) noted that game wardens in Uganda likely under-reported successful instances of humans scavenging on carnivore kills. Fenced reserves, or reserves with strict rules against kleptoparasitism appeared to have reduced (reported) incidents, or absence of incidents. However, where enforcement is lacking, kleptoparasitism has been associated with other unlawful activities i.e., illegal diamond extraction in remote parks in the Central African Republic [20].

In contrast to game meat, people have legal ownership of livestock and viewed repossession of depredated livestock carcasses as ‘taking back’ the meat of an animal they already owned. Thus, prey type is an important factor in assessing kleptoparasitism of carnivore kills because not only do people have the legal right to repossess a livestock carcass, they also may be more motivated to do so from a herding (cultural) standpoint. As a result, both humans and carnivores may face increased risk of injury in confrontations over possession of livestock carcasses.

Culture, religion, and social status influenced whether communities or individuals condoned kleptoparasitism. Therefore, it is possible that, within the range states where kleptoparasitism was confirmed, the occurrence was localized or sporadic. For example, meat theft from carnivores is common practice within the nomadic Mbororo in Northern Cameroon [96]. In contrast, Muslims, a prevalent religion in Sudan and Tanzania, hold strict taboos regarding how animals must be slaughtered (i.e., halal) [97], making meat obtained through kleptoparasitism unfit for human consumption. Some viewed kleptoparasitism as socially beneath them, while others who stood to gain a share of the meat were more accepting of the practice. Unsurprisingly, economic status was reported as an important factor; community members with low income, including park staff with low remuneration, were reportedly more likely to partake in the activity often to supplement their protein supply. Because people who kleptoparasitize carnivores gain an immediate and tangible benefit i.e., meat to consume or sell for cash, it raises the question as to why this practice is not more ubiquitous. It is possible (and highly likely) that insufficient knowledge even among experts and stakeholders, attributable at least in part to a reluctance among local people to report the practice, resulted in significant underestimates of the frequency with which modern human kleptoparasitism occurs.

### Costs to carnivores

The energetics of a carnivore lifestyle are complex [1, 98]. Comprehensive calculations of the costs for carnivores to locate, pursue, capture, kill, defend, lose, and subsequently replace their prey are beyond the scope of this paper. However, increasingly, new technologies i.e., accelerometer and GPS collars, allow for quantification of the energetics of carnivore locomotion and hunting behavior [4, 5, 7, 8, 99], as well as the behavioral and physiological responses of carnivores to human disturbance [43, 44]. Combined with other measures e.g., prey size, length of time feeding prior to interruption, amount of meat lost, these methods could provide an improved accounting of the costs that modern humans exact by disturbing carnivores for access to their kills.

For lions, costs and benefits of hunting vary across sex and age classes. Individuals perform different roles during a hunt, and once a kill is made, all lions do not feed equally [79]. Lion males may displace females and feed exclusively for a period of time [79]. If humans disrupt feeding and remove prey shortly after it has been killed, especially when entire carcasses are taken, the individuals who participated in the hunt endure the costs while obtaining few benefits. Loss of an entire carcass can occur quickly when people become aware of a fresh kill. An incident witnessed by one of the authors (PAW), involved 2 lionesses and 4 cubs (approx. 3mon old) that scattered in different directions away from the carcass of an adult female kongoni, *Alcelaphus buselaphus*, upon which they had just begun feeding when approached by 5 men brandishing machetes and long poles. The men used the poles to carry away the entire carcass of the kongoni which the lionesses had killed only 20min prior and on which they could, theoretically, have spent > 4hrs feeding if left undisturbed [100]. Loss of large carcasses may particularly impact young cubs because cubs obtain more food when feeding on large carcasses than when feeding on small carcasses [79].

Depending on prey size and number of animals feeding, lions may remain near a kill and return to feed over the course of several days [79]. Thus, scavenging meat when lions are absent does not mean that they had finished feeding. Likewise, prey consumption among solitary felids e.g., leopard, puma, lynx, *Lynx lynx*, is prolonged, with a kill often providing food for multiple days [36, 98, 101]. Human kleptoparasitism may involve a portion of meat only. As recorded in documentary footage [102], Maasai in Kenya targeted lion kills with the intention of taking only a leg. Similarly, a visitor to Waza National Park, Cameroon, was observed to opportunistically take a leg from a freshly lion-killed roan antelope, *Hippotragus equinus*, (de Iongh, pers. obs.). Although losing a partial carcass may be less costly, the amount remaining for successive feeding bouts is reduced [20] and other costs i.e., stress, risk of injury, energetic costs of prey replacement, are incurred.

Energy budgets are influenced by environmental variables including terrain [9], season [103], ambient temperature [104], and geographic region. Where prey density is low e.g., West and Central Africa [90, 91], loss of a kill constitutes a pronounced hardship as carnivores may have to travel greater distances to find another vulnerable prey, thereby increasing their energetic costs [1, 6, 92, 100]. Greater travel distance also increases both the risk of encountering territorial conspecifics and, for lionesses with small cubs, the amount of time that young are left unaccompanied [79].

Carnivores chased from their kill are subject to injury by the people who displace them. Confrontational scavenging may involve hand-held weapons e.g., machetes, clubs, hurled rocks and sticks, and shouting (White, pers. obs.). On multiple occasions in protected areas in Zambia, groups of local men carrying machetes and clubs directly approached a concealed research vehicle that was broadcasting sounds of a dying buffalo meant to attract lions. Upon discovering the sound source, the men proffered the explanation that “if we had found lions, we would not have stolen their kill” even though no inquiries had been made as to their intentions (White, pers. obs.).

When humans remove entire carcasses, scavenging species also lose food resources [36]. Where resources are sparse, utilization of carcasses, including bone consumption, increases and can result in higher rates of tooth breakage and wear (gray wolf, *Canis lupus*, [105]). Spotted hyenas in the Luangwa Valley (LV) and Greater Kafue Ecosystem (GKE), Zambia, two regions where human kleptoparasitism was reported to occur frequently (this study), were found to have the highest rates of tooth breakage among any hyenas sampled [10]. High rates of human kleptoparasitism on lion kills that reduced scavengers’ access to carrion may have contributed to the high rates of tooth wear and fracture found in LV and GKE spotted hyenas. Carrion consumption is an important, largely understudied, component of energy transfer in food-webs [106–108]. Human removal of carrion from ecosystems can result in cascading ecological effects on scavenger communities [51–54].

While this paper focuses on direct impacts of human kleptoparasitism on carnivores, this practice also presents a significant concern in the context of ’One Health’ [109], which is a unifying framework integrating health of people, animals, and the environment. The majority of zoonotic emerging infectious diseases originate in wildlife [110–112]. Human kleptoparasitism represents a human-wildlife interface which may increase the likelihood of zoonotic disease transmission [111] both through the handling (butchering) and consumption of wild game i.e., bushmeat [113, 114]. Bushmeat is known to harbor diseases potentially transmissible to humans [115], although the health risks have not been thoroughly investigated [114, 116–118].

That people risk confronting dangerous large carnivores on their kills highlights the lack of available protein in many marginalized communities [35, 116, 118]. Despite accounts of kleptoparasitism being easily accomplished [80], there are numerous reports of large carnivores (lion [19, 34], leopard [19], and tiger, *Panthera tigris*, [55]) attacking and killing people in defense of their prey.

Often, however, lions and other large carnivores do not attempt to defend their kills when challenged by humans, especially when people approach in groups [20, 79, 102]. The opportunity to obtain meat from a kill may increase human tolerance for living near large carnivores [119, 120]. For example, in Uganda, local people benefitted from the predictability of kills made by a particular lion and a leopard with two cubs whom they routinely drove off kills to obtain meat [19]. While any increase in tolerance towards carnivores might be viewed as a measure of conservation success, repeated disturbance and meat and carcass theft are clearly detrimental to carnivores. Human kleptoparasitism on lions in the Gir Forest, India, is believed to have directly contributed to the lion population decline [33, 121]. Likewise, the female leopard in Uganda repeatedly robbed of its kills ultimately attacked its human tormentors in efforts to maintain possession of its prey to feed itself and two cubs [19]. Thus, the short-term benefit of tolerance must be weighed against the cumulative, long-term costs to individual carnivores and local carnivore populations. Ensuring that people have access to alternative sources of protein, thereby eliminating their need to obtain meat from carnivore kills, is key to addressing this issue.

## Conclusion

Our survey found modern human kleptoparasitism on kills made by lions, and possibly other large carnivores in Africa, to be geographically more widespread than previously reported. Humans disrupted carnivores at their kills of wild game and livestock to procure game meat and compensate for losses, respectively. Economic hardship was a main driver of the practice, with game meat important both for personal consumption and trade. Although modern human kleptoparasitism may appear inconsequential in comparison to other anthropogenic threats, the frequency at which carnivores lose kills to humans is likely underestimated. The fact that our surveyed experts repeatedly cited a lack of information, and the strong likelihood that cases go unreported, suggests that the impacts of modern human kleptoparasitism may be higher than are currently recognized. Whether chased or scavenged, the loss of meat or entire carcasses to humans, particularly wild game, represents additive costs to carnivores that should not be dismissed. We argue that human kleptoparasitism of carnivore kills poses an important yet largely cryptic anthropogenic threat that negatively impacts not only the individual carnivores that lose their meal, but also scavenger populations and the broader ecological community. Further, the potential health threats to humans from taking meat from carnivore kills have not been investigated. We propose that modern human kleptoparasitism warrants greater scrutiny.

## Supporting information

S1 Checklist Inclusivity in global research questionnaire. (DOCX)

S1 Appendix Survey questionnaire. Survey on humans taking meat from kills made by lions (kleptoparasitism).
(DOCX)

S1 Table Current African lion range states and survey status for this study. (DOCX)
