# Supplementary material for: Human procurement of meat from lion (Panthera leo) kills: Costs of disturbance and implications for carnivore conservation
Source: PLoS One. 2024 Aug 14;19(8):e0308068. doi: 10.1371/journal.pone.0308068 (PMC11324114; doi:10.1371/journal.pone.0308068)
Supplement: S1 Appendix — Survey on humans taking meat from kills made by lions (kleptoparasitism). (DOCX) [file pone.0308068.s002.docx]

**Supplemental Appendix 1: Survey Questionnaire**

Survey on humans taking meat from kills made by lions (kleptoparasitism)

The information you provide in this questionnaire will be treated with utmost confidentiality and used for academic purposes only. Please reply for all areas for which you have information.

1) Please list the country(s) and area(s) in which you currently (or previously) have conducted lion research or fieldwork and how many years you have been in each area

Area years

__________________________________ _________

__________________________________ _________

2) Do you have any knowledge of humans taking meat from lion kills in your study area? Y/N (if multiple areas are covered, please specify per area)

If NO, please skip to Question # 9

If YES, was your knowledge obtained through:

a. Direct observation or evidence

b. Reliable reports

c. Local gossip

d. Other (please explain) ____________________________________

3) To the best of your knowledge, how was the meat obtained? (if multiple areas are covered please specify per area)

a. Actively chasing lions from a kill

b. Scavenging kill remains while the lions were not in the immediate area

c. Humans responding to researcher-produced playback sounds intended to attract lions

d. Do not know

e. Other (please explain) _____________________________________

4) Did this activity involve: (if multiple areas are covered please specify per area)

a. Local residents

b. Non-residents (Transient/Nomadic/Refugee/Seasonal workers)

c. Game Scouts

d. Do not know

5) Do these activities appear to be: (if multiple areas are covered please specify per area)

a. Opportunistic

b. Deliberate e.g., targeting/following lions when they hunt

c. Do not know

6) How many times have you heard of humans taking meat from lions in the area? (if multiple areas are covered, please specify per area)

a. 1-5 times

b. 5-10 times

c. More than 10 times

d. It does occur in the area, but do not know how often

7) Would you say that the frequency of this occurrence is: (if multiple areas are covered, please specify per area)

a. Staying the same over time

b. Increasing

c. Decreasing

d. Do not know

8) Do you think that humans taking meat from lion kills is: (if more areas are covered, please specify per area)?

a. Infrequent and does not represent a serious threat to lions

b. Frequent enough to potentially have a negative impact

c. Increasing in frequency and a serious concern for lions

d. Not enough information to say if there is an impact or not

e. Do not know

f. Other (please explain) ______________________________________

9) In general, do you believe that humans taking meat from lion kills represents a serious threat to lion populations even if you have not observed it in your study area? Y/N (if multiple areas are covered, please specify per area)

10) Do you have any knowledge of humans taking meat from other predators? (if multiple areas are covered, please specify per area)

a. Leopard

b. Hyena – spotted/brown/striped

c. Cheetah

d. Wild dog

e. Does not occur in this area

f. Do not know

11) Is this practice of taking meat from predators: (if multiple areas are covered, please specify per area)?

a. Accepted culture in the area

b. Taboo but people do it anyway

c. No real cultural significance either way

d. Do not know

e. Other: (please explain) _________________________________

12) Are there any comments or details that you wish to add?

Thank you very much. All responses will be strictly confidential and anonymous.
