# Supplementary material for: Human procurement of meat from lion (Panthera leo) kills: Costs of disturbance and implications for carnivore conservation
Source: PLoS One. 2024 Aug 14;19(8):e0308068. doi: 10.1371/journal.pone.0308068 (PMC11324114; doi:10.1371/journal.pone.0308068)
Supplement: S1 Table — (DOCX) [file pone.0308068.s003.docx]

**Supplemental Table 1. Current African lion range states^1^ and survey status for this study.**

| *Region* | *Country* | *Surveyed* | *Total # Responses (surveys and e-mailed inputs)* | *Human Kleptoparasitism Confirmed* |
| --- | --- | --- | --- | --- |
| East Africa | Ethiopia | No | n/a | n/a |
| East Africa | Kenya | Yes | 14 | Yes |
| East Africa | Tanzania | Yes | 10 | Yes |
| East Africa | Somalia | No | n/a | n/a |
| East Africa | Uganda | No | n/a | n/a |
| West Africa | Benin | Yes | 2 | Yes |
| West Africa | Burkina Faso | Yes | 1 | Yes |
| West Africa | Niger | Yes | 1 | Yes |
| West Africa | Nigeria | Yes | 1 | No |
| West Africa | Senegal | Yes | 1 | No |
| Central Africa | Cameroon | Yes | 3 | Yes |
| Central Africa | Central African Republic | No | n/a | n/a |
| Central Africa | Chad | No | n/a | n/a |
| Central Africa | Congo (DRC) | No | n/a | n/a |
| Central Africa | South Sudan | No | n/a | n/a |
| Central Africa | Sudan | Yes | 1 | No |
| Southern Africa | Angola | No | n/a | n/a |
| Southern Africa | Botswana | Yes | 5 | Yes |
| Southern Africa | Mozambique | Yes | 3 | Yes |
| Southern Africa | Namibia | Yes | 1 | Yes |
| Southern Africa | South Africa | Yes | 10 | Yes |
| Southern Africa | Zambia | Yes | 5 | Yes |
| Southern Africa | Zimbabwe | Yes | 1 | Yes |

^1^ Nicholson et al. 2023.
